# New Partnerships for Co-delivery of the 2030 Agenda for Sustainable Development

**DOI:** 10.1007/s13753-020-00293-8

**Published:** 2020-08-10

**Authors:** Adrian Bucher, Andrew Collins, Ben Heaven Taylor, David Pan, Emma Visman, James Norris, Joel C. Gill, John Rees, Mark Pelling, Marta Tufet Bayona, Sonia Cassidy, Virginia Murray

**Affiliations:** 1 UK Collaborative on Development Research, London, NW1 2BE UK; 2 Disaster and Development Network (DDN), Department of Geography and Environmental Sciences, Northumbria University, Newcastle-upon-Tyne, NE18ST UK; 3 Evidence Aid, Abingdon, Oxfordshire OX13 5DB UK; 4 Global Health, Medical Research Council Swindon, Swindon, SN2 1FL UK; 5 Department of Geography, King’s College London, London, WC2B 4BG UK; 6 VNG Consulting Limited, Kent, TN24 8DH UK; 7 Ordnance Survey, Southampton, SO16 0AS UK; 8 Global Geoscience, British Geological Survey, Keyworth, NG12 5GG UK; 9 Earth Hazards and Observatories, British Geological Survey, Nottingham, NG12 3EN UK; 10 ALLFED, London, UK; 11 Global Disaster Risk Reduction, Public Health England, London, SE1 8UG UK

**Keywords:** 2030 Agenda for sustainable development, Disaster risk reduction, DRG, International development, Partnerships, Research funding

## Abstract

Partnerships have become a corner stone of contemporary research that recognizes working across disciplines and co-production with intended users as essential to enabling sustainable resilience-building. Furthermore, research that addresses sustainable development challenges brings an urgent need to reflect on the ways that partnerships are supported, and for the disaster risk management and resilience communities, efforts to support realization of the wider 2030 Agenda for sustainable development bring particular pressures. In November 2019, the UK Disasters Research Group (DRG) brought together a number of key stakeholders focused on disaster risk, resilience, and sustainability research relevant to Official Development Assistance to consider how fit for purpose existing partnership models are for the pace of change required to deliver the priorities of the wider 2030 Agenda. Participants were invited to discuss how research partnerships across three levels (individual and project-based; national and institutional; and international) could be improved based on elements that facilitate robust partnerships and learning from aspects that hinder them. From the discussions, participants emphasized the importance of effective communication mechanisms in building partnerships, co-designing projects, and establishing shared objectives. Enhanced approaches to addressing equitable partnerships and funding more substantive timelines will be key to responding to the challenges of the 2030 Agenda.

## Introduction

Partnerships have become a cornerstone of the delivery of contemporary resilience-focused research. Approaches that support multi-, trans-, or interdisciplinary research and co-production with intended users are recognized as essential to research that can effectively strengthen resilience and support sustainable development. In disaster-affected states, particularly in low and middle income countries (LMICs), partnerships can also ensure that research agendas are more relevant to local contexts, drive innovation, convene decision-making spaces, and support research infrastructure to generate evidence-based policy to build resilience and respond to disasters.

Partnerships among researchers are increasingly looked for by research funders, to bring together resource to confront challenges of sustainable development. These challenges are dynamic and bring a need to reflect on the ways that partnerships are supported and deployed in research. For the disaster risk management and resilience communities, ensuring address of the priorities articulated in the 2030 Agenda for sustainable development (UN 2015), the Sendai Framework for Disaster Risk Reduction 2015–2030 (UNISDR 2015), and the Agenda for Humanity,^1^ alongside meeting climate-related goals, brings both challenges to and opportunities for innovative partnerships.

In November 2019, the Disasters Research Group (DRG) brought together key UK stakeholders from across academia, government, civil society, think tanks, and the public sector focused on Official Development Assistance (ODA) relevant disaster risk, resilience, and sustainability research to explore new partnerships for delivery of the wider 2030 Agenda.

The DRG is a forum convened by the UK Collaborative on Development Research (UKCDR) comprising senior representation from 20 organizations in the UK that fund and/or support disaster risk reduction research including the Department for International Development, UK Research and Innovation (UKRI), and Wellcome, as well as national representatives of several international bodies such as the United Nations Office for Disaster Risk Reduction (UNDRR), the World Meteorological Organization, and the World Health Organization (UKCDR 2020).

The aim of the DRG is to enhance research and technology-based disaster risk reduction through improved coordination of research funders, providers, and users to deliver components of the UK’s commitment to disaster risk reduction. The group also serves to guide the direction of disaster risk reduction funding. This is informed by reviewing trends in the emerging research landscape, which is largely achieved through active engagement with the UK Alliance on Disaster Research (UKADR)—an independent network of UK research institutions, mainly universities active in ODA-focused disaster research, associated with the Global Alliance of Disasters Research, GADRI (UKADR 2017).^2^

Since its establishment in 2009, the DRG has played an influential role in discussions surrounding disaster risk resilience at both the national and international levels and was credited by UNDRR as one of the most influential bodies in framing the role of science and technology within the Sendai Framework.

There are four major demands placed on existing partnership models by the complexity and interconnectedness of global challenges inherent in the 2030 Agenda.^3^ Research should:

- meet urgent needs;
- respond to emergent phenomena;
- enable equity in partnerships—both internal to project teams (partnering researchers and decision makers across levels) as well as with external stakeholders; and
- be people-centered, not justified or led by innovation in technology alone.

Participants reflected on these demands by assessing three levels of partnerships (project-based, institutional and national, and international) to identify those factors that work well, those that work less well, and how future partnerships could be improved. While the discussions stemmed from a diversity of thought from across the disaster research space, it is acknowledged that the findings largely capture perspectives from those in the Global North and that discussions on the development of future partnerships would greatly benefit from a range of perspectives of stakeholders in LMICs.

## Individual and Project-Based Partnerships

Driven by the commitment of researchers, individual and project-based partnerships offer much in the form of flexibility, cost-effectiveness, and defined timelines which, in the context of disaster risk management and resilience research, has several advantages. For instance, rapid research responses can be mobilized quickly under this partnership model to fill knowledge gaps in an emergency situation and inform future resilience and potentially life-saving strategies.

### What Works Well

When reflecting on the elements that work well in individual and project-based partnerships, participants highlighted the ease of relationship building, compared to the complex partnerships commonly associated with working in large consortia. In particular, participants discussed how healthy individual and project-based partnerships enable more effective and agile communication mechanisms that can help identify areas of cooperation from the outset of a research project, facilitate creative discussions, and more easily establish a shared vision. With the opportunity to take advantage of such communication mechanisms, project-based partnerships were especially valued for their ability to convene like-minded individuals with shared interests and foster an environment of trust and respect among researchers.

### What Works Less Well

In discussions among participants on those areas that work less well in project-based partnerships, many were quick to highlight the issue of partnerships being insufficiently equitable. From the way that much UK funding systems work, most grants are typically awarded to, and managed by, a designated lead institution, predominantly a UK university, which in turn disburses funds to their partners. This presents a number of challenges to overseas partner institutions, such as being paid in arrears and the requirement for funding to be channeled through the coordinating UK-based lead institution rather than via a project specific manner. This can have significant implications on research in terms of how far in advance research can be planned, the approaches used, and overall quality of outputs. These implications were said to be especially magnified for partnerships involving institutions based in LMICs.

Further elaborating on the theme of insufficient equity, participants highlighted how project-based partnerships and associated funding tend to be concentrated around a small number of LMIC-based institutions. As a result, research misses out on knowledge generated by LMIC institutions with limited opportunities to partner with UK institutions, while those that are frequently engaged become overcommitted and overburdened on projects that often overlap with each other. This congestion for some LMIC institutions, and failure of others to be recognized, was thought to be partially a result of a lack of understanding by UK institutions on the incentives that drive LMIC-based partners to take on numerous projects despite lacking capacity, as well as lack of effective coordination among UK institutions, donors, and program management bodies. It also reflects the need to find mechanisms to broaden awareness among LMIC researchers of research opportunities, and to expand networking to grow the pool of potential partnerships across LMIC and UK institutions.

Project-based partnerships were also sometimes criticized for affording insufficient mechanisms for those people at risk and for whom resilience-building initiatives are intended to inform research prioritization, with agendas more reflective of external, preexisting funder and academic interests. There remains limited engagement with the existing knowledge of those agencies already engaging with at-risk populations. In the disaster risk and resilience space, future-focused approaches could be greatly improved if more opportunities would be afforded to those groups likely to be most directly affected by risks to inform the research agenda and inform monitoring and evaluation processes. This should be extended to vulnerable populations and those experiencing post-disaster recovery, as well as those agencies working to support these populations.

### How Future Partnerships Can Be Improved

In addressing issues around equity, participants suggested building the capacity of LMIC partners to develop, lead, and manage research projects while also making proposal application processes for UK funding more accessible through language and call requirements.^4^ Participants also welcomed recent initiatives by a selection of UK research funders to fund international partners directly.

More broadly, participants underlined the significance of being explicit about the intended respective benefits of partnership. Greater equity can be supported through partners from the outset of a project jointly developing a shared vision, an agreed set of deliverables, a common understanding of what each partner wants to gain from a specific collaboration and how the benefits will be shared beyond the end of funding. Participants also highlighted the importance of developing more comprehensive impact frameworks that recognize social, environmental and economic, benefits, as well as the intangible benefits of new partnerships, altruism and individual motivation.

Communication around shared learning about the benefits and approaches was also said to play a crucial role in planning to bring forward research outputs from collaborative projects. Specifically, it was emphasized that the knowledge built up by key individuals in project-based partnerships can be shared through establishing networks among colleagues and communities of practice. Furthermore, early-career researchers from one project may be future research leaders and, through career progression, the interdisciplinary approaches from earlier projects may be adopted more widely. This will, in turn, require reviewing pathways for career progression, ensuring incentives that recognize the value of engaging in collaborative research that delivers tangible benefits for partnering at risk groups and those agencies that support them.

## Institutions and National Partnerships

Research partnerships at the institutional and national level are characterized by their ability to facilitate collaborations that draw from multidisciplinary expertise across institutions and the experiences of different sectors to exchange ideas and address complex challenges. Disaster risk reduction strategies have become increasingly informed by research generated from such partnership models, benefiting from the insights of a diverse range of partners such as from the financial sector, civil society, and government agencies.

### What Works Well

For partnerships at the institutional and national level, all groups underlined the importance of understanding and accepting the political, economic, and social situations where research takes place and the complexities of working in partnership with national institutions (which can be facilitated by diverse partnerships involving local policy intermediaries to help mediate the relationship between researchers and policymakers). This particularly applies to those instances involving government stakeholders as there may be challenges in using research and evidence to feed into policy debates in light of overlapping or competing mandates. In disaster-affected communities, there remains a need to consider how research is shared with participating populations, as research in these settings is at particular risk of being viewed as extractive.

Successful partnerships were therefore said to put a lot of emphasis on establishing clarity on the motivations and languages of different agencies, including between policymakers, researchers, and the media, and in identifying a common set of goals.

### What Works Less Well

In terms of what works less well, participants noted that institutional and national partnerships can be adversely impacted by a variety of exogenous influences. These include institutions viewing UK funding schemes as a means to pursue commercial interests rather than bring about long-term change, as well as pressure from research funders to build institutional partnerships in short funding cycles.

As a result, participants noted that these can lead to the formation of partnerships based on questionable motivations that are unable to achieve sustainable outcomes. Examples of these included top-down collaborations where inter-institutional partnerships are based on personal rather than professional relationships and, at a higher level, a duplication of efforts by groups of institutions to produce research using slight variation in approaches used.

### How Future Partnerships Can Be Improved

Given the challenges in developing institutional and national partnerships, there were discussions on the role that research bodies and funders could play in facilitating matchmaking between different agencies. It was thought that the existence of such a platform could make partnership building easier as it could help, among other things, identify common approaches used by institutions and opportunities (and demand) to conduct interdisciplinary research.

Suggestions were also made on the possibility of having longer-term UK funding frameworks whereby small funding pools could be designated for longer-term and/or non-quantifiable components of disaster research (particularly institutional partnership building) where research outcomes are expected to be deferred outside of the standard 3–5-year project timeline.

## International Partnerships

Extending the institutional partnership model to a global level, international partnerships are able to take advantage of an even larger community of knowledge and expertise to develop interdisciplinary research to address complex challenges. In addition to becoming more frequent in recent years (due to advances in air travel and communication technologies), international partnership models allow the disaster risk resilience community to benefit from evidence generated by LMIC institutions, provide opportunities for research capacity strengthening, and can lead to the establishment of regional knowledge hubs.

### What Works Well

Despite the amount of time required to build robust partnerships, participants praised the ability of the disaster risk and resilience research community to identify key people and organizations to work with when forming international partnerships. In particular, multi-sectoral partnerships that bring together the academic, public, private, and non-governmental organization sectors were seen as effective tools to stimulate innovation. This was seen to largely be driven by the effective integration of stakeholder mapping into project design (rather than at later stages) to ensure that the right partners are involved, assumptions are challenged, and priorities are agreed.

Effective communications during the co-design and co-production of research projects was especially highlighted by participants since this is crucial to the success of international and multi-sectoral partnerships as the careful selection of the project focus, including the phrasing of objectives, can help to bring together partners and helps to remove some of the politics from research.

### What Works Less Well

On those elements of international partnerships that work less well, participants noted that while there is a demand for (rapid) international partnerships, the resource investment needed to build such partnerships is often lacking. This was exemplified using the case of UK-led international partnerships involving LMIC-based institutions when there is little to no follow-on funding available to grant holders to develop partnerships, resulting in scarcity of long-term capacity-building programs. Similarly, participants lamented the lack of prioritization and support of UK funding schemes to support partnerships between LMIC institutions.

While participants noted that the research community is aware of cultural issues, an improved understanding of cultural norms and sensitivities would allow the community to be more effective. With international partnerships, it was said that there can be a tendency to homogenize groups (for example, academia, communities) and overlook the complexity of communities and the impacts of this on policy and implementation. This was said to be the case where organizations may be looking to create partnerships in new and emerging systems of governance.

### How Future Partnerships Can Be Improved

To improve international partnerships, participants spoke of the need to decolonize UK research and to continue the push for equitable partnerships by ensuring that partners in LMICs have a greater input in shaping research priorities based on the needs of those countries and communities that research is seeking to help. This would involve giving those partners greater control over resource allocation, which in turn would also help address continued issues relating to capacity building. It was proposed that such shifts could be facilitated by broader application of processes of change in funding mechanisms.

For instance, mentors could support LMIC-based partners in identifying relevant funds and funding criteria and the designing of projects, including by ensuring that they have access to the relevant information in a format and language appropriate to their needs. To that end, participants highlighted the important role of trusted brokers to facilitate the research process as key in driving forward the research impact of increasingly complex international partnerships.

While there has been a greater awareness of these issues as a result of the transparency commitments attached to increasing ODA funding to support research in recent years, participants noted that this has resulted in a complex funding landscape with limited access to some institutions not considered to serve a core academic function. In response to this, it was suggested that there could be a better understanding and awareness of the different sources of funding available and funders could make it easier for a wider group of practitioners to access and help to influence the nature of this research funding.

## Forward Look

The urgent action required to address global challenges has only increased in recent years and progress towards the delivery of the wider 2030 Agenda has become more reliant on rigorous research facilitated by effective partnerships. COVID-19 and the economic consequences are likely to see reduced budgets for research and this places even greater importance on understanding how to manage partnerships to enable research that can be timely, adaptive, fair, and people-centered. While members of the UK disaster risk, resilience, and sustainability research community have developed important understanding of some of the components and mechanisms vital to enabling effective and agile partnership models, especially in terms of establishing shared objectives, the sustainability of partnerships and partnership processes are considered a cause for concern. A key way forward will be to develop research frameworks that support and value more equitable forms of partnership and resource more substantive timeframes that enable complex research partnership approaches to flourish.

## Footnotes

1 https://www.agendaforhumanity.org/.

2 Examples of such engagements include a combined DRG and UKADR event in July 2019 on “New Points of Departure in Transitioning Disaster Reduction and Sustainability Challenges” attended by 130 people from across the disaster risk reduction research landscape.

3 See https://tomorrowscities.org/tomorrows-cities-fit-2030-agenda.

4 Funders can play an important role in setting the tone for equitable partnerships and there exists some practical guidance on this. See Dodson (2017) and KFPE (2018).
